# Driving Forces of Dynamic Changes in Soil Erosion in the Dahei Mountain Ecological Restoration Area of Northern China Based on GIS and RS

**DOI:** 10.1371/journal.pone.0142331

**Published:** 2016-03-16

**Authors:** Xiao Li, Xiang Niu, Bing Wang, Peng Gao, Yu Liu

**Affiliations:** 1 Shandong Agricultural University, College of Forestry, Taishan Mountain Forest Ecosystem Research Station, Tai'an, Shandong 271018, China; 2 Research Institute of Forest Ecology, Environment and Protection, Chinese Academy of Forestry, Beijing 100091, China; University of Vigo, SPAIN

## Abstract

Dynamic change in soil erosion is an important focus of regional ecological restoration research. Here, the dynamic changes of soil erosion and its driving forces in the Dahei Mountain ecological restoration area of northern China were analyzed by LANDSAT TM remote sensing captured via geographic information system (GIS) technologies during three typical periods in 2004, 2008 and 2013. The results showed the following: (1) a decrease in intensive erosion and moderate erosion areas, as well as an increase in light erosion areas, was observed during two periods: one from 2004 to 2008 and the other from 2008 to 2013. (2) Between 2004 and 2008, the variation in the range of slight erosion was the largest (24.28%), followed by light erosion and intensive erosion; between 2008 and 2013, the variation in the range of intensive erosion area was the largest (9.89%), followed by slight erosion and moderate erosion. (3) Socioeconomic impact, accompanied by natural environmental factors, was the main driving force underlying the change in soil erosion within the ecological restoration area. In particular, the socioeconomic factors of per capita forest area and land reclamation rate, as well as the natural environmental factor of terrain slope, significantly influenced soil erosion changes within the ecological restoration area.

## Introduction

Soil erosion, a global environmental concern, restricts human survival and development [1, 2] and has received widespread attention [3, 4]. Soil erosion is the process of soil transfer within a specific time and place and is influenced by both natural factors and human activity [5]. Many countries have increasingly engaged in scientific efforts to solve this problem. Researchers have systematically studied factors such as types of soil erosion, amounts of soil erosion, and changes in soil erosion and have attempted to simulate and predict this process [6, 7].

To study changes in soil erosion and the conditions under which it occurs, researchers have studied the formation and change of global soil erosion from ancient times to the present and have summarized efforts to restore and conserve soil and water, such as social economic aspects: terracing, reforesting, constructing dams and agriculture size, migration, etc.[8,9]. Experts have attempted to study changes in soil erosion using advanced scientific methods, such as the Morgan model, SWAT model and the Universal Soil Loss Equation (*USLE*), which are often used to simulate the soil erosion process and to estimate the amount of soil erosion [10–12]. Furthermore, the geochemical mass balance approach is used to analyze and compare the effects of human agricultural activities and natural factors on soil erosion in different locations [13]. Researchers have also studied changes in the physical and chemical properties of soil [14] to uncover different soil erosion processes and to analyze the factors underlying soil erosion [15]. With the development of information technology in recent years, it has become possible to study patterns of spatial change in soil erosion and their driving forces over different periods of time using remote sensing (RS) and geographic information system (GIS) technologies [16]. Thus, significant breakthroughs in uncovering the driving mechanisms of erosion, based on both natural factors and socio-economic factors, have been reported. For example, researchers studying site conditions driving erosion in the Upper Nam Wa watershed of Northern Thailand used RS and GIS to learn that the absolute majority of total soil loss could be attributed to shifting cultivation along a steep slope, which accounted for approximately 70% of the total soil loss, while other factors, such as site conditions, made comparatively minor contributions [17]. Meanwhile, the methodology for assessing soil erosion, which based on GIS and RS, is also developing [18].

In China, in-depth studies of changes in soil erosion have also taken place. Researchers have probed the relationship between soil erosion changes and changes in land use, as well as the relationship between changes in soil erosion intensity and quantity [19]. Furthermore, they have analyzed the dynamic changes and causes of soil erosion in detail, considering the type, area, intensity and distribution of soil erosion based on RS and GIS [20]. In parallel, temporal and spatial changes in soil erosion were explored, and the impact of climate change and human activity on soil erosion was analyzed [21].

In 1999, the Ministry of Water Resources of China implemented a nationwide Ecological Restoration Project in an ongoing effort to conserve soil and water for many years. The Ministry explores methods of controlling soil erosion depending on ecological self-reparative capabilities under different natural and socioeconomic conditions by probing dynamic changes in soil erosion and its driving forces over different time periods to provide a scientific basis for the rational allocation and quantitative evaluation of the repair efficiency of the Ecological Restoration Project [22]. At present, much of this research is dedicated to evaluating the technologies of the Ecological Restoration Project and their benefits [23–25]. However, few studies have characterized changes in soil erosion and its underlying causes in ecological restoration areas, and it is difficult to accurately assess the efficiency of soil and water conservation efforts in mountainous areas. Therefore, the ecological restoration area of Dahei Mountain in Liaoning Province, a typical hilly region experiencing soil erosion in the northern part of China, was chosen to study dynamic changes in soil erosion. Changes in this hilly ecological restoration area were quantitatively analyzed by soil erosion classification, changes in the rate of soil erosion intensity, transfer matrix, and other indicators, based on LANDSAT TM remote sensing images captures during three typical periods in 2004, 2008 and 2013 using geographic information system (GIS) technologies. The driving forces of soil erosion were evaluated by multiple stepwise regression analysis in the study area.

## Materials and Methods

The project area setup, observation indicators and test methods were all based on the Specifications for Assessment of Forest Ecosystem Services in China (LY/T 1721–2008), Indicators System for Long-term Observation of Forestry Ecosystems (LY/T 1606–2003) and Observation Methodology for Long-term Forest Ecosystem Research (LY/T 1952–2011) [26].

### Ethics Statement

The research station used in this study is managed by Shandong Agricultural University. The study was approved by the Taishan Mountain Forest Ecosystem Research Station of the State Forestry Administration. Moreover, the studied area was not a research base of the Special Fund for Forestry Scientific Research in the Public Interest, was not a national park or other protected area of land or sea, and did not affect endangered or protected species.

### Study area

**Fig 1:**
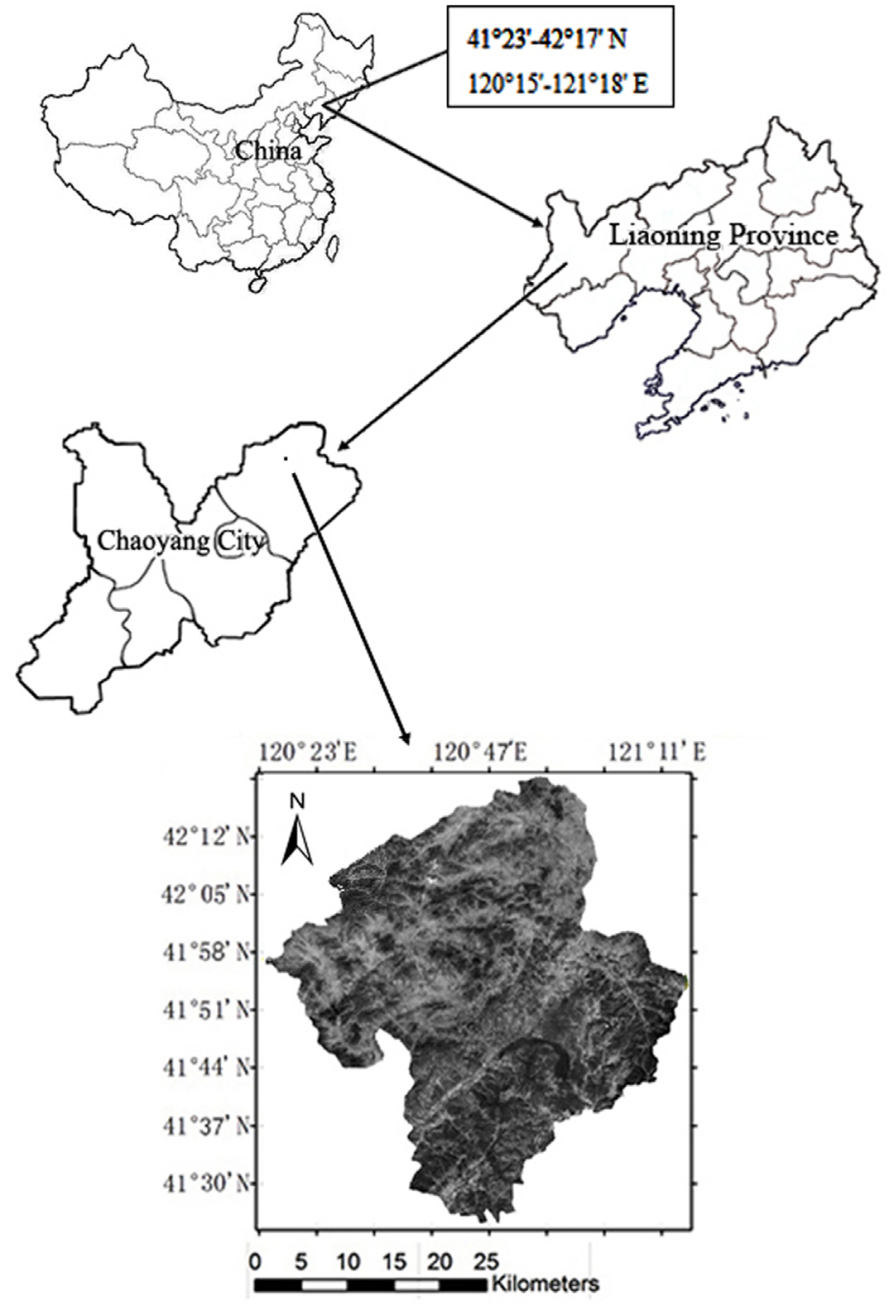
Map of the location of the study area.

The study was conducted in Chaoyang City, within Liaoning Province (120°15'-121°18'E, 41°23'-42°17'N), and the total experimental area encompassed 4464 km^2^. The study site was located in the hilly area in western Liaoning and the middle reaches of the Daling River (Fig 1), which is typical of hilly regions affected by soil erosion in the northern part of China. Dahei Mountain is the highest peak in the study area, with an altitude of 1074 m. The region has a semi-arid, semi-humid continental monsoon climate with an average temperature of approximately 8°C and an annual average rainfall of 410–550 mm, over 80% of which falls between June and August. The vegetation region is the northern boundary of the North China flora, and it lies at the convergence of the flora of North China and Inner Mongolia. As such, it lies within the East Asian summer green deciduous broad-leaved forest [27]. The soil type is mainly leached cinnamon, which is similar to the American soil classified as Usdalfs.

### Data Sources

Based on RS software (Erdas Imagine 8.7) and GIS (ArcGIS 9.3) technology, Landsat TM remote sensing images of three typical periods in 2004, 2008 and 2013 (Namely: on June 16, 2004; on June 29, 2008; on June 25, 2013. and multispectral image resolution is 30 m, the path/row is 122 /36) were processed. To reduce errors in image processing, the data were geometrically corrected by image sharpening and cloud removal, and areas of interest were drawn to carry out human-computer interaction translations according to hue, saturation, shape, shadow, texture, position, and size. We performed a comprehensive analysis and correction of the translation results using topographic, geological, and soil maps, as well as current land utilization data and a combined GNSS (Global Navigation Satellite Systems) survey of the location. Thus, soil erosion within the ecological restoration area at Dahei Mountain was characterized to analyze erosion dynamics.

### Research methods

Indicators of soil erosion, such as soil erosion classification, rate of erosion intensity change, a transfer matrix of soil erosion intensity, and dynamic changes in soil erosion were analyzed, and its causative factors were analyzed by multiple stepwise regression analysis.

#### Soil erosion classification

Water erosion is the main type of erosion within the study area. Landsat TM remote sensing images of three typical periods in 2004, 2008 and 2013 were used as the main data source, and rainfall, land use, vegetation coverage, and soil type data, as well as topographic and other thematic maps, were used to derive an average soil erosion intensity map for the study area in accordance with the Revised Universal Soil Loss Equation (*RUSLE*) [28, 29]. According to the standards of the Chinese Ministry of Water Resources for the classification and gradation of soil and water erosion (SL190-2007), as well as observations of the study area, soil erosion data from the study area were divided into four soil erosion intensity types: slight erosion, light erosion, moderate erosion and intensive erosion.

#### The change rate of soil erosion intensity (R)

The rate of change in erosion intensity within a specific time period can be described quantitatively to compare differences in soil erosion intensity and to predict trends in soil erosion [30]. The equation used to calculate *R* is as follows:

$$R=\frac{U_{b}-U_{a}}{U_{a}}\times \frac{1}{T}\times 100\%$$ (1)

Where *R* is the change in soil erosion intensity during the study period; *Ua* and *Ub* represent the early stage and final phase of soil erosion intensity, respectively; and *T* is time in years.

#### The transfer matrix of soil erosion intensity

Due to the influence of natural factors, social development, and human activity, all types of soil erosion intensity were condensed into one matrix. The dynamic transfer matrix describes the conversion among all types of soil erosion intensity [31, 32] and can be used to simulate the process of soil erosion in the hilly ecological restoration area and to form a matrix describing the dynamics of soil erosion. Combined with the Landsat TM data from this region, we can detail a period of time during the transformation of various soil erosion intensity types.

#### Multivariate stepwise regression analysis

Multivariate stepwise regression analysis is performed as follows [33]:

Y=B+0BX1+1BX2+2⋅⋅⋅+BXmm

Where *Y* is the random variable. According to the principle of generalized least squares (GLS), we can calculate the undetermined coefficient (*B*_0_, *B*_1_, *B*_2_, …, *B*_*m*_) in the formula using *n* (*i* = *1*, *2*, *3*, …, *n*) groups of observed values (*X*_1i_, *X*_2i_, …, *X*_mi_, *Y*_i_). On this basis, multivariate stepwise regression analysis individually brings the independent variables into the regression equation from large to small according to the effect of the independent variable on *Y*, and those independent variables that do not exert a significant effect on *Y* are not included in the regression equation.

To comprehensively determine the driving force underling the dynamic changes in soil erosion in the study area, this study selected 9 driving force indicators (two indicators for natural environmental factors and seven for socioeconomic factors; Table 1) known to influence erosion in the ecological restoration area of Dahei mountain for constructing an evaluation index system corresponding to the driving force behind soil erosion in the study area. We selected the soil erosion area ratios (the percentage of soil erosion area over total area) of 29 townships in the study area as dependent variables and every evaluation index as an independent variable and performed a stepwise regression analysis by the “Stepwise” method in SPSS 19.0. We then established a multiple stepwise regression model of soil erosion area ratio and driving factors to analyze the dominant driving factors underlying soil erosion dynamic change. In the “Stepwise” method, the criteria were “Probability-of-F-to-enter = 0.050, Probability-of-F-to-remove = 0.100” [34].

**Table 1:** Evaluation index system of the driving force factors of soil erosion.

The evaluation index				Units of drivingforce factors	Source of the data
**Natural factors**	1	*X*1	annual precipitation	mm	Meteorological data of Bureau of meteorology of Chaoyang City
	2	*X*2	terrain slope	°	Data of Landsat TM RS images from 2004, 2008 and 2013
**Social and economic development factors**	1	*X*3	population density	person/km^2^	Data of statistical yearbook of history (2004–2013)and investigation
	2	*X*4	land reclamation rate	%	Data of statistical yearbook of history(2004–2013) and investigation
	3	*X*5	per capita cultivated land area	hm^2^/ per capita	Data of statistical yearbook of history(2004–2013) and investigation
	4	*X*6	per capita forest area	hm^2^/per capita	Data of statistical yearbook of history(2004–2013) and investigation
	5	*X*7	per capita amount of stock raising	kg/per capita	Data of statistical yearbook of history(2004–2013) and investigation
	6	*X*8	per capita agricultural output value	yuan/per capita	Data of statistical yearbook of history(2004–2013) and investigation
	7	*X*9	per capita grain output	kg/per capita	Data of statistical yearbook of history(2004–2013) and investigation
**Soil erosion change index**	1	*Y*	soil erosion area ratio	%	Data of Landsat TM RS images from 2004, 2008 and 2013 and Data of statistical yearbook of history (2004–2013) and investigation

## Results and Analysis

### Dynamic changes in soil erosion area

Table 2 shows the change in soil erosion intensity in the ecological restoration area of Dahei Mountain during different periods. From 2004–2008, the erosion area was 2833.3 km^2^. In 2008, this accounted for 63.47% of the total area of ecological restoration, which decreased 894.14 km^2^ compared with 2004 (20.03% erosion area in 2004). Light erosion area increased by 520.5 km^2^, accounting for 11.66% of the total area; the moderate erosion area decreased by 370.52 km^2^, accounting for 8.3%; and the intensive erosion area decreased by 1044.12 km^2^, accounting for 23.39%. In total, light erosion area increased over this period, while moderate erosion and intensive erosion areas were reduced. Additionally, erosion area overall decreased, and soil erosion intensity decreased.

**Table 2:** Area change of soil erosion intensity in the ecological restoration area during different periods.

Soil erosion intensity type	2004		2008		2013		Area change(km^2^)	
	Area (km^2^)	Proportion (%)	Area (km^2^)	Proportion (%)	Area (km^2^)	Proportion (%)	2004–2008	2008–2013
**Slight erosion**	736.56	16.5	1630.7	36.53	2270.39	50.86	+894.14*	639.69
**Light erosion**	691.03	15.48	1211.53	27.14	1255.72	28.13	520.5	44.19
**Moderate erosion**	1361.97	30.51	991.45	22.21	619.16	13.87	-370.52	-372.29
**Intensive erosion**	1674.44	37.51	630.32	14.12	318.73	7.14	-1044.12	-311.59
**Soil erosion area**†	3727.44	83.5	2833.3	63.47	2193.61	49.14	-894.14	-639.69

^“†”^ In the table, soil erosion area is the sum of light erosion area, moderate erosion area and intensive erosion area

* “+” indicates the increase; “−” indicates the decrease.

The soil erosion area was 2193.61 km^2^ in 2013, which accounted for 47.84% of the total area, and decreased by 639.69 km^2^ compared with 2008 (2833.3 km^2^). During this period, the light erosion area increased by 44.19 km^2^, accounting for 0.99% of the total area; the moderate erosion area decreased by 372.29 km^2^ (8.34%), and the intensive erosion area decreased by 311.59 km^2^, accounting for 6.98%. During this period, erosion area and erosion intensity continued to decrease.

### The change rate of soil erosion intensity

**Fig 2:**
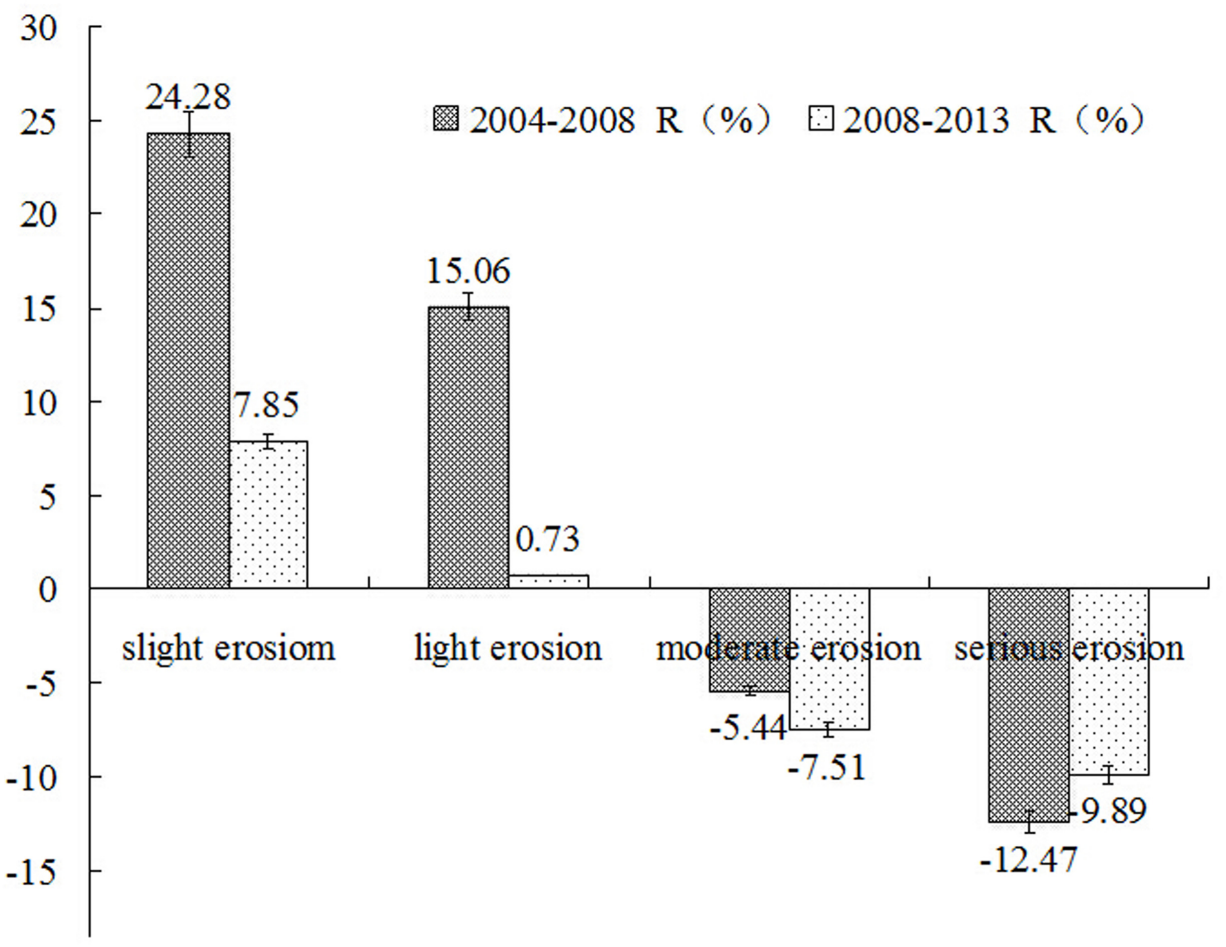
The change rate of soil erosion intensity of the ecological restoration area during different periods.

The average annual change rate (*R*) of all soil erosion intensity types in the study area can be calculated using formula (1) (Fig 2). From 2004–2008, the *R* value of the slight erosion area was the largest, accounting for 24.28% of the change in total soil erosion area, indicating that the change amplitude of the slight erosion area was the largest, followed by those of the light and intensive erosion areas, which accounted for 15.06% and -12.47% of the change in total soil erosion area, respectively. The moderate erosion area exhibited less change, accounting for -5.44% of the change in total soil erosion area. From 2008–2013, the *R* value of the intensive erosion area was the highest, accounting for -9.89% of the change in total soil erosion area, and its change amplitude was the largest, followed by the slight and moderate erosion areas, which accounted for 7.85% and -7.51% of the change in total soil erosion, respectively. The change in the light erosion area was smaller, accounting for 0.73% of the change in total soil erosion area.

### Transformation of soil erosion intensity area at different levels

**Fig 3:**
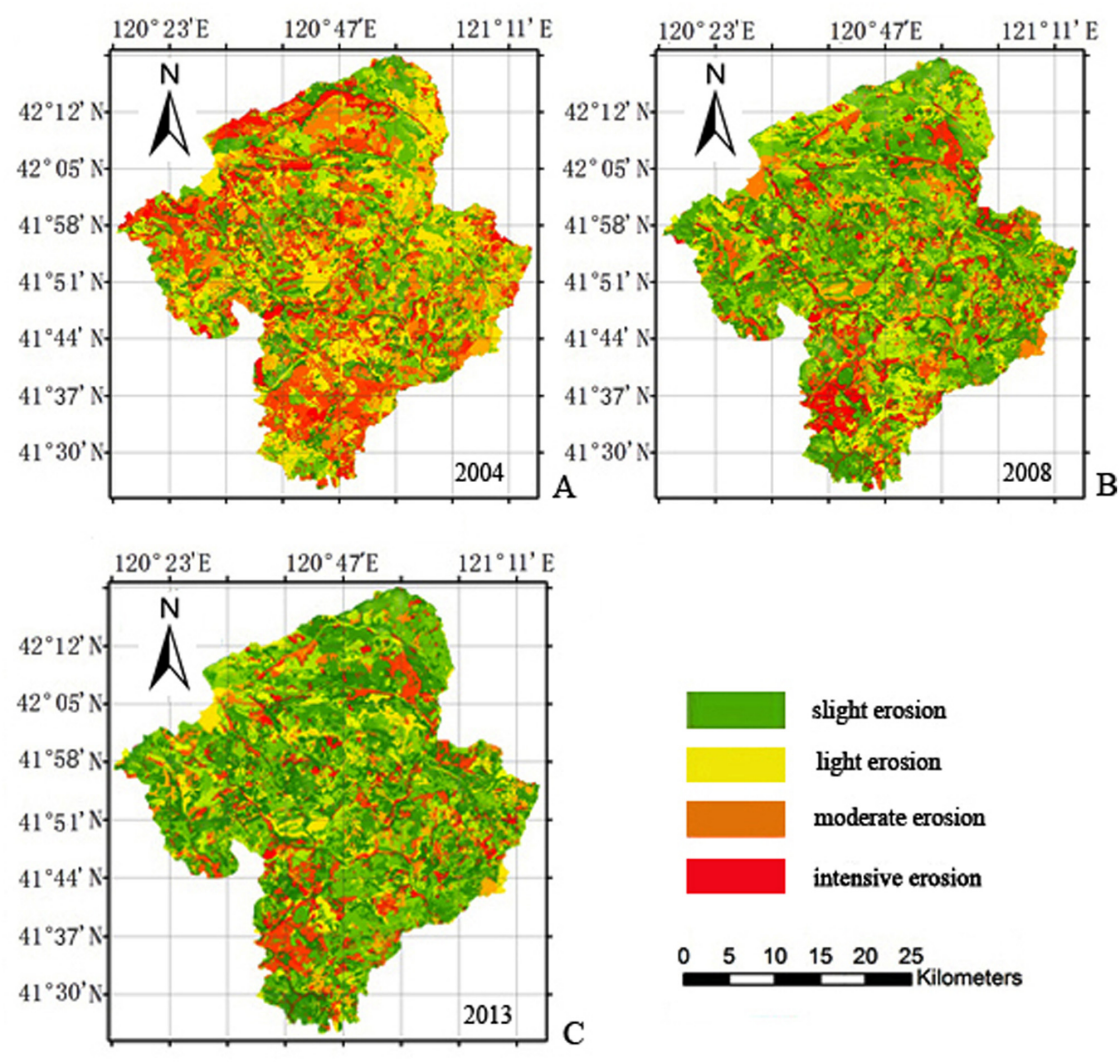
Soil erosion changes atlas in the Dahei Mountain ecological restoration area during different periods.

From 2004–2008, slight erosion was mainly converted from light erosion and moderate erosion (458.46 km^2^ and 449.08 km^2^, respectively), and the intensive erosion areas were converted from 211.59 km^2^ (Table 3; Fig 3A and 3B). Light erosion was mainly converted from moderate erosion and intensive erosion (550.86 km^2^ and 379.88 km^2^, respectively), and a portion of the light erosion area was converted from slight erosion (141.96 km^2^). Intensive erosion was only converted from moderate erosion (38.39 km^2^).

**Table 3:** Transfer matrix of soil erosion of the ecological restoration area during 2004–2008 (km^2^).

Dynamic transfer matrix	2004					2008 Total
		1†	2	3	4	
**2008**	1	511.57	458.46	449.08	211.59	1630.7
	2	141.96	138.83	550.86	379.88	1211.53
	3	83.03	93.74	323.64	491.04	991.45
	4	0	0	38.39	591.93	630.32
**2004 Total**		736.56	691.03	1361.97	1674.44	4464

^“†”^ 1 Slight erosion, 2 Light erosion, 3 Moderate erosion, 4 Intensive erosion.

Based on the transfer matrix of soil erosion area corresponding to 2004–2008, various types of soil erosion intensity changed between 2004 and 2008; some erosion types were transferred into or converted from other types. All types of soil erosion in the left lower triangular matrix were enhanced to varying degrees, and the change area was 357.12 km^2^, accounting for the restoration of 8% of the total area. This mainly reflects an increase in soil erosion caused by unreasonable agricultural development and human destruction within the ecological restoration area. Different soil erosion intensity types in the right upper triangular matrix were reduced to different degrees, and the change area was 2540.91 km^2^, accounting for 56.92% of the total area undergoing ecological restoration. The change data mainly reflects achievements in water and soil conservation efforts. The overall analysis of the transformation in this period indicated a reduction in soil erosion area and in soil erosion intensity. Furthermore, the soil erosion conditions in the ecological restoration area were substantially improved.

From 2008–2013, slight erosion was mainly converted from light erosion and moderate erosion; the change areas were 330.78 km^2^ and 291.50 km^2^, respectively (Table 4, Fig 3B and 3C), and the area that was converted from intensive erosion was 168.74 km^2^. Light erosion was converted from moderate erosion, intensive erosion and slight erosion; the change areas were 389.26 km^2^, 166.95 km^2^ and 151.33 km^2^, respectively. Moderate erosion was converted from light erosion and intensive erosion; the change areas were 234.81 km^2^ and 145.53 km^2^, respectively. Intensive erosion was mainly converted from light erosion and moderate erosion; the change areas were 97.76 km^2^ and 71.87 km^2^, respectively.

**Table 4:** Transfer matrix of soil erosion of the ecological restoration area during 2008–2013 (km^2^).

Dynamic transfer matrix	2008					2013 Total
		1†	2	3	4	
**2013**	1	1479.37	330.78	291.5	168.74	2270.39
	2	151.33	548.18	389.26	166.95	1255.72
	3	0	234.81	238.82	145.53	619.16
	4	0	97.76	71.87	149.1	318.73
**2008 Total**		1630.7	1211.53	991.45	630.32	4464

^“†”^ 1 Slight erosion, 2 Light erosion, 3 Moderate erosion, 4 Intensive erosion.

From the transformation matrix of soil erosion area during 2008–2013, it can be observed that areas of various types of erosion intensity changed over these five years. Similar to the changes observed during 2004–2008, some erosion types not only transferred to other types, but were also converted from other types of soil erosion during this period. The overall degree of different soil erosion types in left lower triangular matrix was enhanced (555.77 km^2^), accounting for 12.45% of the total area undergoing ecological restoration; the degree in the right upper triangular matrix was reduced (1492.76 km^2^), accounting for 33.44% of the total area undergoing ecological restoration. Primary expressions were observed: areas of slight and light erosion in Dahei Mountain increased, while areas of moderate and intensive erosion decreased at this stage. This mainly reflects the decrease in soil erosion intensity and the overall improvement of conditions.

### Driving force factors underlying dynamic changes of soil erosion

This study analyzed the soil erosion area ratios and the leading soil erosion driving factors in 29 townships of the Dahei Mountain ecological restoration area using multivariate stepwise regression analysis methods. We built a multivariate stepwise regression model as follows:

Y=−0.384X+60.463X+40.227X2 (2)

Where *X*_6_ is the per capita forest area; *X*_4_ is the land reclamation rate; X_2_ is the terrain slope; and *Y* is the soil erosion area ratio.

**Fig 4:**
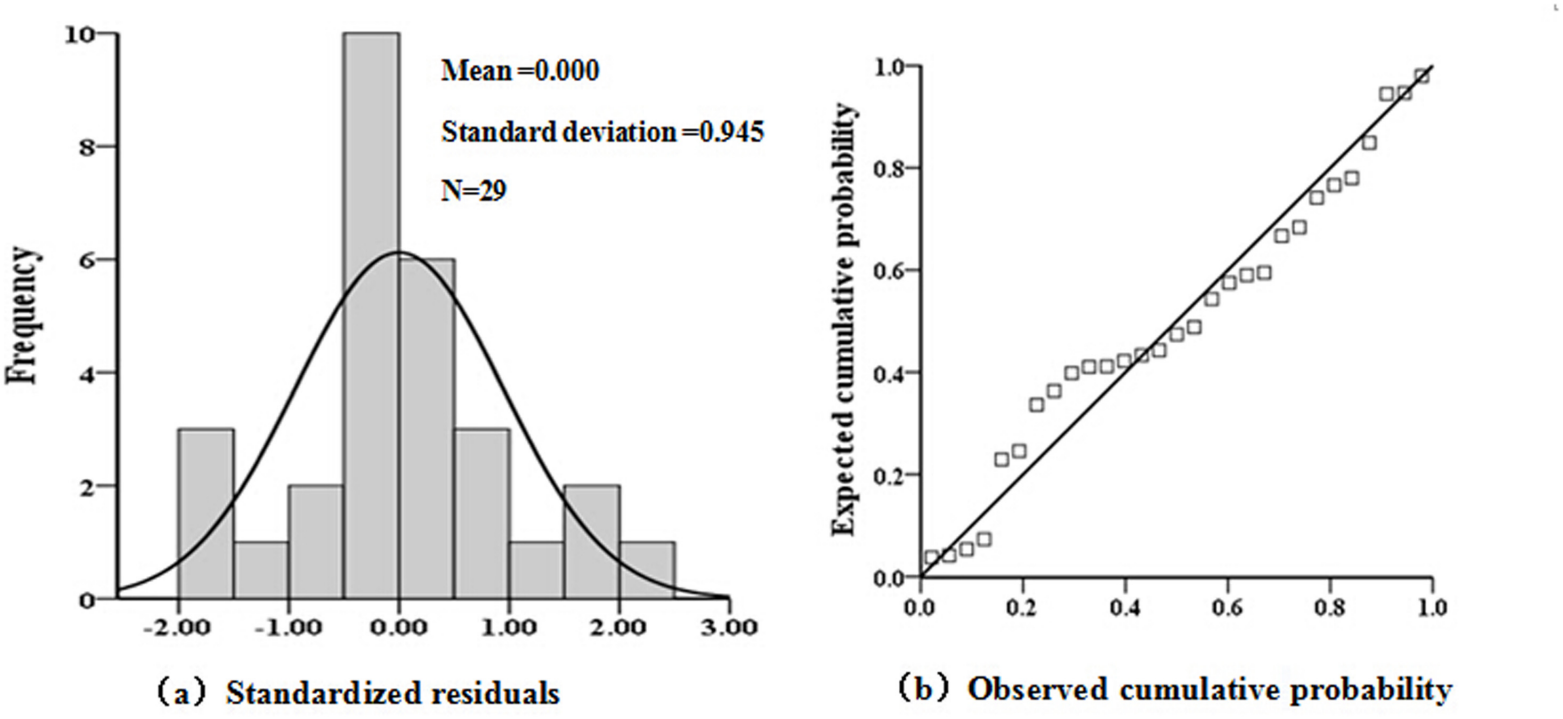
The histogram of standardized residuals (a) and the normal probability plot of standardized residuals (b).

This study performed the F (Table 5) test on the regression model (2) and found that the probability (sig. = 0.000) of the variance analysis was far less than 0.05; therefore, the regression model (2) was significant, with a substantial linear regression effect. From Fig 4, it can be observed that sample residuals were distributed in a nearly normal distribution, and splashes were closely scattered at the slash, corresponding to a normal distribution with a mean of zero. This indicated that the random variable residuals obeyed a normal distribution, and the regression model (2) passed the significance test. Therefore, the regression model (2) can be used to objectively evaluate the relationship between soil erosion area ratio and soil erosion driving factors.

**Table 5:** Significance test and summary table of the multivariate stepwise regression model of soil erosion driving factors in the ecological restoration area.

Model	*F*	Significant level	*R*†	*R*^2^	Adjusted *R*^*2*^	Estimate standard error
**Regression model**	81.852	0.000	0.953	0.908	0.897	0.3217033

^“†”^ *R* indicates the complex correlation coefficient.

From the regression model (2), we know that the soil erosion area ratio is proportional to the terrain slope and the land reclamation rate and is inversely proportional to the area of forest per capita. The complex correlation coefficient R was 0.953 (Table 5), indicating that there are remarkable correlations between soil erosion ratio (*Y*) and per capita forest area (*X*_*6*_), land reclamation rate (*X*_*4*_), and terrain slope (*X*_*2*_). Soil erosion area tended to increase with the improvement of land reclamation rate (*X*_*4*_) and an increase of terrain slope (*X*_*2*_); however, the soil erosion area ratio decreased with the increase of per capita forest area (*X*_*6*_). From the partial correlation coefficient (Table 6), the impact on soil erosion area ratio (*Y*) is *X*_*4*_ > *X*_*6*_ > *X*_*2*_, from large to small. *X*_*6*_ and *X*_*4*_ are socioeconomic factors, and it can be observed that the socioeconomic factors have a primary influence on soil erosion, while *X*_*2*_ (terrain slope), a natural environmental factor, was second. This shows that while natural environment factors may be the foundation of soil erosion, the excessive exploitation of forest resources and land resources are leading factors in soil erosion in the ecological restoration area of Dahei Mountain. It can be observed that the main driving factor of soil erosion in this region is a socioeconomic factor, as per capita forest area and land reclamation rate clearly influence soil erosion.

**Table 6:** Multivariate stepwise regression coefficient of soil erosion driving factors in the ecological restoration area.

Model	Standard error	Standardized coefficient	t	sig.	Partial correlation coefficient
**(Constant)**	0.060		16.402	1.000	
***X***_**6**_	0.100	-0.384	-3.83	0.001	-0.608
***X***_**4**_	0.088	0.463	5.257	0.000	0.725
***X***_**2**_	0.085	0.227	2.683	0.013	0.473

## Discussion

### The effect of ecological restoration engineering on the dynamic changes of soil erosion

Severe soil erosion was a common problem in the ecological restoration area before the initiation of restoration efforts. To some extent, the alleviation of soil erosion is a direct embodiment of the effectiveness of ecological restoration engineering. Studies have shown that ecological restoration projects can effectively improve the use of land and change the original topography, enhancing vegetation coverage and greatly reducing the intensity of soil erosion while drastically reducing soil erosion area [35, 36]. These results are consistent with the results of this study. Our research area was a typical undulated hilly region experiencing soil erosion in the northern part of China. Since the implementation of ecological restoration projects from 2004–2013, soil erosion in this area has greatly decreased. By 2013, the size of the intensive erosion area had reduced to 1355.71 km^2^, and the moderate erosion area had reduced to 742.81 km^2^, while the slight and light erosion areas increased to 1533.83 km^2^ and 564.69 km^2^, respectively. At the same time, the driving force factors (X_6_, X_4_, X_2_) in this study were closely associated with ecological restoration measures. The dynamic changes in soil erosion that were observed were benign due to the implementation of a series of ecological restoration measures in the Dahei Mountain region.

In 2004, prior to the implementation of ecological restoration measures, the cultivated land area was 2195.4 km^2^, and the forest area was 618.7 km^2^, accounting for 49.18% and 13.86% of the total area, respectively. During the ten years from 2004 to 2013, the implementation of the Ecological Restoration Project brought a significant decrease in cultivated land and a significant increase in forest (a decrease of 30.17% and an increase of 106%, respectively). The Ecological Restoration Project included the implementation of ecological restoration engineering in the residual woodlands, as well as forest and grass prohibition to promote recovery. Furthermore, forest and grass at the top and upper portions of the mountain were refreshed, and farmland was returned to forest or fruit while developing economical forests and pastures under a high standard of land use in the middle and middle lower parts of the hillside. Moreover, the efficient afforestation of unused land and wasteland was carried out. Just like the result “land cover is one of the influential factors that affect soil erosion” [37, 38], these projects increased the area of forestland and grassland and improved land cover of the study area, and then the soil erosion area was reduced.

According to the Landsat TM RS image data collected in 2004, 2008 and 2013, as well as the results of other research [39], a large area of sloping farmland in the ecological restoration area was present at a higher slope. From 2004–2013, we engineered and implemented terracing of the cultivated sloping land to achieve a combination of high efficiency economic forest and water conservation tillage. As a result, more sloping farmland areas were returned to forest or fruit within the study area, and the average gradient of arable land was significantly decreased, thus reducing the possibility of soil erosion. In the study area, a comprehensive control project was implemented for the river channel during 2004–2013, thereby increasing the construction of check dams, small reservoirs, and other small water storage projects in the channel, which increased land for water area. These water facilities provided a water source for the implementation of forest and grass engineering, resulting in soil and water conservation and reducing soil erosion.

Above all, the construction and implementation of ecological restoration engineering changed the original topography of the land and improved the coverage of vegetation. It also reduced the intensity and area of soil erosion, and greatly improved the ecological status of the region.

### Driving force analysis of dynamic changes of soil erosion

Natural and socioeconomic factors are two major driving forces of soil erosion. Natural factors include terrain, climate, landform, gradient, slope direction, geology, soil and vegetation, while socioeconomic factors include population, economy, system policy, and technical measures. The results of several studies have shown that while soil erosion and ecological degradation can be attributed to drought and the vulnerability of the ecological environment, the biggest influence was human economic activity [40, 41]. This is consistent with the results of this study. In our study, we found that the implementation of a series of ecological restoration measures, including terracing of cultivated sloping land, conversion of degraded farm land into forest, enclosure of mountain for the increase of greenness, fruit forest construction and construction of water conservancy facilities, significantly improved vegetation and land cover. These measures were reflected in the form of per capita forest area, land reclamation rate, terrain slope in the study. They all significantly influenced soil erosion, and were the dominant driving factors of soil erosion in the Dahei Mountain ecological restoration area. Meanwhile, of the factors driving soil erosion, per capita forest area and land reclamation rate had a greater effect than did terrain slope on the change in soil erosion. Thus, socioeconomic factors (i.e., human activities) are the primary driving factor of soil erosion, while natural environmental factors play a secondary role. This difference in rank was mainly due to the economic structure of the ecological restoration area in Dahei Mountain, which is based on the development of agroforestry. Therefore, this region’s unreasonable level of agriculture, forestry development and construction activities, especially in relation to the overexploitation of forestry resources and land resources, promoted serious soil erosion and ecological degradation. Following this, a situation arose in which social economic factors majorly influenced soil erosion. This illustrates the prominent conflict between local social and economic development and ecology and underscores the need for strengthening ecological protections and rehabilitation management.

## Conclusions

1. This analysis showed that intensiveand moderate erosion areas decreased, while light erosion area increased in the study area from 2004–2008 and 2008–2013.
2. From 2004–2008, the variation range of the slight erosion area was the largest, with an increase of 24.28%, followed by the light and intensive erosion areas; from 2008–2013, the variation range of the intensive erosion area was the largest but had decreased by 9.89%, followed by the slight and moderate erosion areas.
3. Socioeconomic factors, which stem from natural environment factors, were the main driving force behind soil erosion in the Dahei Mountain ecological rehabilitation area. Moreover, the socioeconomic factors of per capita forest area and land reclamation rate, as well as the environmental factor of terrain slope, significantly influenced soil erosion change in the region.

## Supporting Information

S1 File Data set of the paper (PONE-D-15-28386). (XLS)

## References

[1] Yang QY, Xie YQ, Li WJ, Jiang ZC, Li H, Qin XM. Assessing soil erosion risk in karst area using fuzzy modeling and method of the analytical hierarchy process. Environ Earth Sic. 2014; 71: 287–292.

[2] Ren ZP. Evaluation of regional soil erosion dynamic—a case study of JiHe Demonstration Zone. Yangling China: Northwest A&F University, 2009.

[3] Satoshi U. Applicability of satellite remote sensing for mapping hazardous state of land degradation by soil erosion on agricultural areas. Procedia Environmental Sciences. 2015; 24: 29–34.

[4] Zhao XN, Zhang BQ, Wu PT. Changes in key driving forces of soil erosion in the Middle Yellow River Basin: vegetation and climate. Nat Hazards. 2014; 70: 957–968.

[5] Zhang XW, Zhou YM, Li XS, Yuan C, Yan NN, Wu BF. A Review of Remote Sensing Application in Soil Erosion Assessment. Chinese Journal of Soil Science. 2010; 41(4): 1010–1017.

[6] Lv H, Chen SB, Meng ZG, Wang ZJ, Song JH. Large-scale Classified Mapping in Soil Erosion on RS and GIS. Remote Sensing Technology an Application. 2007; 22(6): 715–717.

[7] Lin WH, Wu YG, Mao DH, Yu Y. Research on Evaluation Method of Regional Soil Erosion Based on Naive Bayesian Model. Yellow River. 2007; 29(12): 71–73.

[8] Dotterweich M. The history of human-induced soil erosion: Geomorphic legacies, early descriptions and research, and the development of soil conservation—A global synopsis. Geomorphology. 2013; 201: 1–34.

[9] Bhandari KP, Aryal J, Darnsawasd R. A geospatial approach to assessing soil erosion in a watershed by integrating socio-economic determinants and the RUSLE model. Nat Hazards. 2015; 75:321–342.

[10] Sanjay KJ, Sudhir K, Jose V. Estimation of Soil Erosion for a Himalayan Watershed Using GIS Technique. Water Resources Management. 2001; 15: 41–54.

[11] Mosbahi M, Benabdallah S, Boussema M.R. Assessment of soil erosion risk using SWAT model. Arab J Geosci. 2013; 6:4011–4019

[12] Naqvi HR, Mallick J, Devi LM, Siddiqui MA. Multi-temporal annual soil loss risk mapping employing Revised Universal Soil Loss Equation (RUSLE) model in Nun Nadi Watershed, Uttrakhand (India). Arab J Geosci. 2013; 6:4045–4056

[13] Yoo K, Fisher B, Ji J, Aufdenkampe A, Klaminder J. The geochemical transformation of soils by agriculture and its dependence on soil erosion: An application of the geochemical mass balance approach. Science of the Total Environment. 2015; 521–522: 326–335. 10.1016/j.scitotenv.2015.03.084 25847176

[14] Wang SJ, Ruan HH, Wang B. Effects of soil microarthropods on plant litter decomposition across an elevation gradient in the Wuyi Mountains. Soil Biology & Biochemistry. 2009; 41: 891–897.

[15] Nacinovic MGG, Mahler CF, Avelar AD. Soil erosion as a function of different agricultural land use in Rio de Janeiro. Soil & Tillage Research. 2014; 144: 164–173.

[16] Arekhi S, Niazi Y, Kalteh AM. Soil erosion and sediment yield modeling using RS and GIS techniques: a case study, Iran. Arab J Geosci. 2012; 5:285–296.

[17] Krishna BKC. Mapping soil erosion susceptibility using remote sensing and GIS: a case of the Upper Nam Wa Watershed, Nan Province, Thailand. Environ Geol. 2009; 57: 695–705.

[18] Kachouri S, Achour H, Abida H, Bouaziz S. Soil erosion hazard mapping using Analytic Hierarchy Process and logistic regression: a case study of Haffouz watershed, central Tunisia. Arab J Geosci. 2015; 8: 4257–4268.

[19] Fan JR, Chai ZX, Liu SZ, Tao HP, Gao P. Dynamical Changes of Soil Erosion in Lizixi Catchment of Sichuan Province by Remote Sensing and Geographical Information System. Journal of Soil and Water Conservation. 2001; 15(4): 25–28.

[20] Wu YH. Analysis on the Dynamic Change and the Causes of Soil Erosion in Ningxia Hui Autonomous Region. Arid Zone Research. 2004; 21(3): 259–262.

[21] Niu RQ, Du B, Wang Y, Zhang LP, Chen T. Impact of fractional vegetation cover change on soil erosion in Miyun reservoir basin, China. Environ Earth Sci. 2014; 72: 2741–2749.

[22] Zheng YL, Gao P, Zhang LY, Wang B. Changes of Land Use and Its Driving Forces of the Ecological Restoration Area in Dahei Mountain in the West of Liaoning Province. Journal of Mountain Science. 2014; 32(6): 691–697.

[23] Yu XX, Niu JZ, Xu JL. Research on the Ecological Restoration in Mountain Watershed. Science of Soil and Water Conservation. 2004; 2(1): 4–10.

[24] Jellinek S, Rumpff L, Driscoll DA, Parris KM, Wintle BA. Modelling the benefits of habitat restoration in socio-ecological systems. Biological Conservation. 2014; 169: 60–67.

[25] Jin JS, Wang RS, Li F, Huang JL, Zhou CB, Zhang HT, et al Conjugate ecological restoration approach with a case study in Mentougou district, Beijing. Ecological Complexity. 2011; 8: 161–170.

[26] Wang B, et al Observation methodology for long-term forest ecosystem research. Beijing: State Forestry Administration 2011; 133–162.

[27] Yang HL, Gao PL, Wang HW, Ding FJ, Dai WG. Characteristics of soil particles fractal dimension under different forest stands of the ecological restoration area in Dahei Mountain area. Science of Soil and Water Conservation. 2009; 7(5): 52–57.

[28] Rozos D, Skilodimou H.D, Loupasakis C, Bathrellos GD. Application of the revised universal soil loss equation model on landslide prevention. An example from N. Euboea (Evia) Island, Greece. Environ Earth Sci. 2013; 70(7): 3255–3266, 10.1007/s12665-013-2390-3

[29] Pradhan B, Chaudhari A, Adinarayana J, Buchroithner MF. Soil erosion assessment and its correlation with landslide events using remote sensing data and GIS: a case study at Penang Island, Malaysia. Environ Monit Assess. 2012; 184:715–727. 10.1007/s10661-011-1996-8 21509515

[30] Duan WX. Study on dynamic changes of soil erosion in Erhai Basin. Science of Soil and Water Conservation. 2011; (11): 38–40.

[31] Zhuang DF, Liu JY. Study on The Model of regional Differentiation of Land Use Degree in China. Journal of Natural Resources. 1997; 12(2): 105–111.

[32] Andrade RFS, Pinho STR. Critical exponents for the long-range Ising chain using a transfer matrix approach. The European Physical Journal B. 2006; 50: 33–37.

[33] Zang SY, Na XD, Li Y, Feng ZK. Driving force mechanism of grassland degradation in Daqing Region, northeastern China. Journal of Beijing Forestry University. 2007; 29(Supp. 2): 216–221.

[34] Pijanowski BC, Brown DG, Shellito BA, Manik GA. Using neural networks and GIS to forecast land use changes: A land transformation model. Computers, Environment and Urban Systems. 2002; 26: 553–575.

[35] Hu SW, Zhou Y, Lv XL. Effect of Land Use Change on the Soil Erosion for the Ecological Recovery Region of DaYao Country. Yunnan Geographic Environment Research. 2006; 18(3): 40–43.

[36] Liu X, Yao XY, Yuan L (2008) Effects of different measures of soil and water conversation on soil erosion in rocky mountainous area of the middle of Shandong Province. Science of Soil and Water Conservation. 2008; 6 (5): 27–31.

[37] Alkharabsheh MM, Alexandridis TK, Bilas G, Misopolinos N, Silleos N. Impact of land cover change on soil erosion hazard in northern Jordan using remote sensing and GIS. Procedia Environmental Sciences. 2013; 19, 912–921.

[38] Wijitkosum S. Impacts of land use changes on soil erosion in Pa Deng sub-district, adjacent area of Kaeng, Krachan National Park, Thailand. Soil Water Res. 2012; 7(1), 10–17.

[39] Lv XZ, Yu XX, Fan DX, Li QY. Estimation of non-point source pollution loads caused by soil erosion in China. Journal of Food, Agriculture & Environment. 2012; 10(2): 1045–1050.

[40] Na B. Research on mechanism of ecological deterioration and protection countermeasures in upper and middle Dang River. Grassland and Turf. 2008; (2): 13–18.

[41] Meng GC, Ma DG, Wang SG, Xie H, Zhang GX. Land eco-environment problems and its driving forces in the upper reaches of the Minjiang River. Arid Lang Geography. 2007; 30(5): 759–765.

